# The Efficacy of Squalene in Cardiovascular Disease Risk-A Systematic Review

**DOI:** 10.3390/nu12020414

**Published:** 2020-02-05

**Authors:** Nurul ‘Izzah Ibrahim, Syed Fairus, Mohamed S. Zulfarina, Isa Naina Mohamed

**Affiliations:** 1 Pharmacoepidemiology and Drug Safety Unit, Department of Pharmacology, Faculty of Medicine, Universiti Kebangsaan Malaysia Medical Centre, Jalan Yaacob Latif, Bandar Tun Razak, Cheras 56000, Kuala Lumpur, Malaysia; 2 Malaysian Palm Oil Board (MPOB), No. 6 Persiaran Institusi, Bandar Baru Bangi, Kajang 43000, Selangor, Malaysia

**Keywords:** squalene, cardiovascular disease, cholesterol, hyperlipidemic, atherosclerosis

## Abstract

Introduction: Cardiovascular disease (CVD) is the leading cause of death worldwide. Squalene (SQ), an intermediate for the cholesterol biosynthesis, has been proposed to act similarly to statins via inhibition of 3-hydroxy-3-methylglutaryl coenzyme A (HMG-CoA) reductase in the liver. Purpose: This paper explores the effects of SQ in CVD. Methods: A systematic review of the literature was performed to identify relevant studies about SQ and CVD. A comprehensive search in Medline and Scopus for relevant studies published between the years 1946 and 2019 was performed. The main inclusion criteria were that the study was published in English; that the study reported association or effect of SQ and CVD; and that CVD should be related to lifestyle variables, aging, or experimentally induced conditions. Results: The literature searches identified 5562 potentially relevant articles, whereby 21 studies met the inclusion criteria. There were three human studies and 18 animal experimental studies included in this paper. Only one human study reported positive outcome of SQ in CVD. The remaining two studies reported inconsistent and/or no effect. For animal studies, 15 studies reported positive effect while the remaining reported negative and/or no effect of SQ on various related parameters. Conclusions: This evidence-based review emphasizes the potential of SQ being used for cardiovascular-related diseases. The effect of SQ, especially of plant-based warrants further exploration. Controlled human observational studies should be performed to provide comprehensive evidence.

## 1. Introduction

Cardiovascular disease (CVD) can be defined as a group of diseases involving the heart and blood vessels. It is the leading cause of death worldwide, accounting for more than 17 billion deaths per year (30% of all deaths) [1,2]. Generally, cardiovascular system is susceptible to many chronic diseases such as myocardial infarction (MI), atherosclerosis, and cardiomyopathies. CVD is the number one silent killer in the world for men and women as they may not be aware of the disease until they experienced a heart attack/MI [3]. Several non-modifiable and modifiable risk factors for CVD are being progressively identified. Male gender, advanced age (>50 years), and family history are among non-modifiable risk factors. Modifiable risk factors include smoking, obesity, sedentary lifestyle, diabetes, high cholesterol or abnormal blood lipids, and hypertension [3]. The incidence of CVD is expected to rise in the next decade due to the aging population and increased pervasiveness of modifiable risk factors [4].

Elevated low-density lipoprotein cholesterol (LDL-C) is a well-known factor that contributes to the major cardiovascular risk factor for CVD incidence [5]. Previous literature has shown that the prevalence of CVD can be reduced by lowering LDL-C through the use of lipid-lowering agents, mainly statins [6,7,8]. Statins are the most effective and widely prescribed drugs currently available for hypercholesterolemia treatment and for managing patients with increased cardiovascular risk [9,10]. Statins act as the competitive inhibitors of 3-hydroxy-3-methylglutarylcoenzyme A (HMG-CoA) reductase at the initial step in cholesterol synthesis, inhibiting the conversion of HMG-CoA to mevalonate [11,12]. Several clinical trials have demonstrated their safety and efficacy in high-risk patients. Among them are HPS (Heart Protection Study of cholesterol-lowering with simvastatin) [13], AVERT (Atorvastatin versus Revascularization Treatment) [14], and PLANET I and II (renal effects of atorvastatin and rosuvastatin in proteinuria patients with and without diabetes) [14]. Although the beneficial effect of the statins is well known, their side effects should not be ignored, ranging from benign and asymptomatic to severe and organ-threatening, especially on liver and kidney function. Despite their benefits, statins have been associated with serious adverse reactions including muscle damage [15], renal failure [16], liver dysfunction [17], and polyneuropathy [10,18]. Alternatively, statins are used in combination with other agents including ezetimibe with clinical efficacy and safety for optimizing LDL level in high CVD risk subjects and statin-intolerant patients [19]. However, using ezetimibe alone or in combination with statins has been associated with the incidence of muscle symptoms [20,21]. Proprotein convertase subtilisin/kexin type 9 (PCSK 9) inhibitors, a new injectable lipid lowering drug, has withdrawn attention due to its attractive mechanism, documented safety trials, and robust LDL-lowering effect. Nonetheless, high therapy costs may limit its use [22,23]. These evidences showed that medicines currently used for lipid lowering have side effects and are costly, suggesting the need to search for alternative therapies with fewer or no side effects. As an alternative, natural products have been considered as a tremendous productive source for new medicines and supplements. For instance, red yeast rice has been reported for its high tolerability and safety profile and has recently been suggested as a lipid-lowering dietary supplement for the general population as well as statin-intolerant patients [24]. Other natural products should be explored for providing alternatives to statin-intolerant patients.

**Figure 1:**
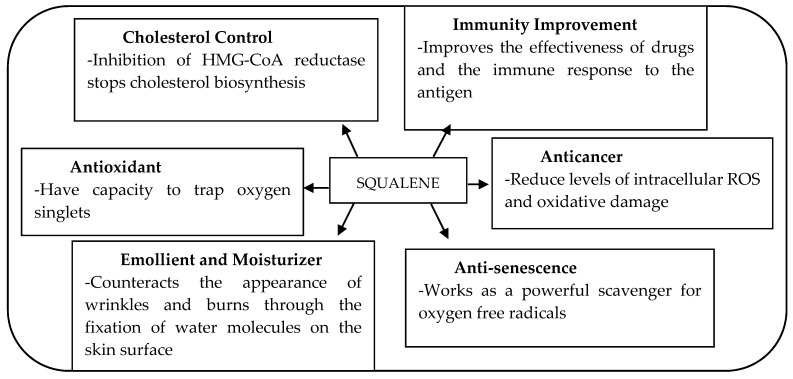
Potential beneficial effects of squalene and the underlying mechanisms responsible for the effects [32,33]; 3-hydroxy-3-methylglutaryl coenzyme A (HMG-CoA).

**Figure 2:**
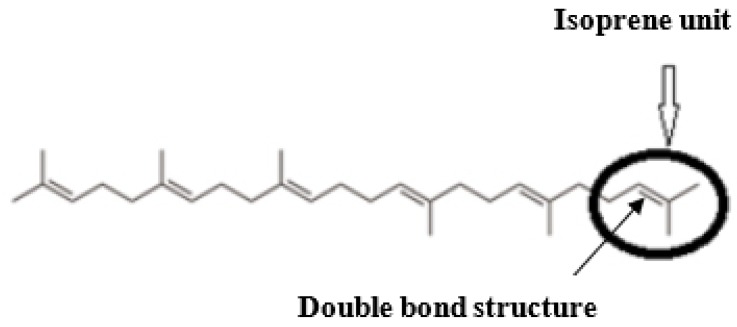
Chemical structure of Squalene (SQ).

As an intermediate in the biosynthesis of phytosterol and cholesterol, it is thus relevant to investigate the effects of squalene (SQ) that might be effective in lowering circulating cholesterol and LDL-C. Evidence of SQ having beneficial effects against CVD revealed that the mechanism was similar to that of statins, which was associated with the inhibitory activity on HMG-CoA reductase in the liver, and downregulates the conversion from acetyl CoA to cholesterol [25]. SQ has been also discovered to possess a wide spectrum of biological functions (Figure 1) such as preventing cell deterioration, being anti-senescence, and improving immunity as well as sexual function [26,27]. In 1916, Tsujimoto Mitsumaru, a Japanese chemist, successfully isolated the SQ (C_30_H_50_) from shark liver oil *(Squalus spp*.) [28,29] and found that the compound was a highly unsaturated isoprenoid hydrocarbon containing six double bonds (Figure 2) [30]. The presence of this double bond structure enabled the isoprenoid hydrocarbon to act as a strong antioxidant and natural antibiotic. Additionally, this biochemical structure has made SQ extremely reactive in getting into the oxidized form, where it binds with the hydrogen ions from water and releases three unbound oxygen molecules, developing into its saturated form squalane, C_30_H_62_. Consequent to this reaction, the oxygen reaches the cells to intensify cellular metabolism, thus enhancing the function of certain organs like liver and kidney [31].

The use of shark as a source of SQ is limited by the animal protection regulations and the presence of organic pollutants such as organochlorine pesticides, polycyclic aromatic hydrocarbons, dioxins, or carcinogenic heavy metals [34]. Hence, extracting SQ from other natural sources especially of plant origin is vital, especially with the increase in SQ demand. SQ is present with a reasonable amount in plant oils such as olive oil, palm oil, amaranth oil, wheat germ oil, and vegetable oil [35]. Compared to other common vegetable oils, palm fatty acid distillate (PFAD), which is a by-product of physical refining of palm oil, contains high amount of SQ up to 415 ± 5 ppm [36] and potentially becomes one of the most valuable sources of squalene. Percentages of SQ content in olive, wheat germ, and rice bran oils range, however, only from 0.1–0.7% [37]. SQ is also present in the human body, secreted by the sebaceous glands to protect the skin by forming 10–14% of lipids in sebum [38]. It is also present in internal organs such as the liver and small intestine at much lower concentrations compared to the skin [28,29,39].

In humans, oral intake of SQ is absorbed at rates of 60 to 85%. Oral uptake and the intestinal de novo synthesized SQ are transported by chylomicrons into circulation and are followed by liver uptake prior to conversion into sterols and bile acids [40] or are resecreted into the bloodstream [41]. Hepatic SQ either biosynthesized or dietary is secreted into very-low-density lipoproteins (VLDL) and low-density lipoproteins (LDL) and is distributed to various tissues. Thus, SQ concentration in plasma lipoproteins represents an equilibrium from dietary intake and intestinal or liver synthesis [42]. Nevertheless, the SQ’s role on plasma lipids in humans and animal models is still unclear due to the controversial observations from several studies including the elevation of plasma cholesterol following SQ intake in rats [43] and hamsters [44] as well as unchanged levels in serum cholesterol among humans [45]. There were also positive observations on SQ supplementation in reducing plasma cholesterol in animals [46,47,48] and humans [49]. Thus, these discrepancies on the effects of SQ on the plasma cholesterol level clearly warrant further investigations. The objective of this current evidence-based review is to explore any original research articles that determined the effects of SQ in CVD, in terms of population/sample, intervention, study design, and outcomes. The review also includes other parameters including antioxidant status, diagnostic marker enzymes, and atheroma lesions that contribute to CVD.

## 2. Methods

### 2.1. Literature Review

A systematic review of the literature was performed to identify relevant studies on SQ and CVD. In conducting a comprehensive search of scientific journals, Medline via Ovid Medline and Scopus published between 1946 to May 2019 were used. The search strategy involved a combination of the following two sets of keywords: (i) squalene and (ii) cardiovascular OR coronary OR heart OR vascular OR *lipid* OR *cholesterol* OR *triglycerid* OR atherosclero* OR stroke OR myocardial infarction OR isch*mi*. During searching, an asterisk (*) was used as a truncation sign to broaden the search to include various word endings.

### 2.2. Selection of Research Articles

The results were restricted to full research articles published in English. Studies that complied with these following inclusion criteria were included: (i) the study reported the association or effects of SQ and CVD and (ii) CVD should be related to lifestyle variables, aging, or experimentally induced conditions. Studies were excluded if i) CVD was related to congenital or other pathological changes; (ii) the article was a review, letter, newsletter, editorial, or case study, and (iii) the study was a duplicated study.

### 2.3. Data Extraction and Management

Papers included in this review were selected based on three phases. Firstly, the papers that did not fulfill the inclusion criteria based solely on their titles were excluded. Secondly, the abstracts of the remaining papers were screened to exclude a second group that did not match the inclusion criteria. Thirdly, the remaining papers from the second phase were scrutinized to exclude any paper that did not match the inclusion criteria. The remaining papers were then screened by at least two reviewers prior to data extraction. Any discrepancies that arose were settled by discussions between the reviewers. The data from the studies were recorded in terms of (i) type of study, (ii) description of sample/population, (iii) description of the method used, (iv) description of the results, and (v) outcomes and comments on the study.

## 3. Results

### 3.1. Search Results

The literature searches identified 5562 potentially relevant articles. Two reviewers independently assessed all articles for inclusion and exclusion criteria based on the title and abstract. A total of 66 articles were retrieved for further assessment and data extraction. Following these assessments, 41 of the articles were excluded due to redundancy between the two databases (*n =* 19); not involving SQ or CVD (*n =* 12); not being primary studies (*n =* 6); being in other languages (*n =* 3); and type of article (*n =* 1).

**Figure 3:**
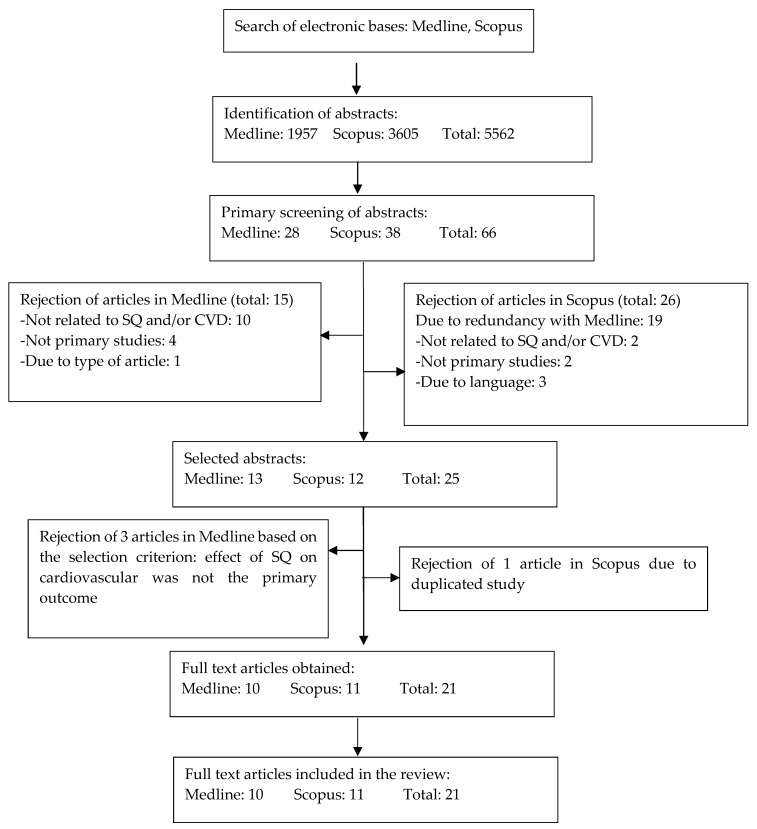
Flow chart to show the selection process of the articles in this review.

Discussions regarding the inclusion and exclusion criteria of the full articles were frequently conducted to resolve differences in opinion between both reviewers. Four articles from the remaining 25 articles were excluded based on the inclusion and exclusion criteria. A total of 21 articles was eventually included for making of this review. The process of paper selection from identifying relevant articles to selecting articles based on the inclusion and exclusion criteria is summarized in Figure 3.

### 3.2. Study Characteristics

The characteristics of animal studies were divided into four: studies related to hyperlipidemia (Table 1), studies related to atherosclerosis (Table 2), studies related to myocardial infarction (Table 3), and studies related to cardiotoxicity and cardiomyopathy (Table 4). Meanwhile, the characteristics of human studies were presented in Table 5. The effects of SQ and CVD has been recorded since the year 1954 until 2016, with the majority conducted within the past 20 years. Three human studies and 18 animal studies were included. In animal studies, different species were used as their study model: rabbits (*n =* 1), hamsters (*n =* 4), mice (*n =* 3), and rats (*n =* 10). The study population for human studies was very different from each other. The study populations in Strandberg et al. [45] and in Miettinen and Vanhanen [50] were less than 20 participants, while Chan et al. [49] had a larger study population of 102 participants. Participants enrolled were diagnosed with hypercholesterolemia with mean age of >50 years and >65 years for Miettinen and Vanhanen study [50] and for Chan et al. [49], respectively. Participants in the study by Strandberg et al. [45] were patients suffering mainly from cerebrovascular or cardiovascular disorders with different degrees of hypercholesterolemia. Participants in Chan et al. [49], however, received pretreatment conditions to avoid carryover effects where a 2-month discontinuation of any previous treatment with lipid-lowering drugs and a 4-week lead-in administration of a single-blind placebo were performed.

All animal studies used male animals. However, Guill’en et al. [56] used both sexes to observe whether SQ modulates atherosclerotic lesion in a sex-dependent manner or vice versa. The experimental hypercholesterolemic condition was induced with high fat or cholesterol diet in six animal studies [44,47,48,52,53,54]. Experimental myocardial infarction using isoproterenol injection was performed in six animal studies [57,58,59,60,61,62]. Moreover, doxorubicin injection was used in Preobrazhenskaya et al. [64] for cardiomyopathy induction while cyclophosphamide injection was used in Motawi et al. [63] for cardiotoxicity induction. Three studies have used genetically engineered mice (i.e., Apo A, Apo E knockout, and KK-Ay obese/diabetes model) [46,52,56]. Guill´en et al. [56] used Apo E knockout mice as these mice may develop severe atherosclerosis on a regular low-fat/low cholesterol diet [65]. SQ was supplemented for different periods. The longest duration of squalene supplementation was in a human study with a 20-week period [49]. In an animal study, the most common duration of SQ treatment was four to six weeks via force-feeding [44,48,51,52,53,54,57,58,59,60,62]. In contrast, the shortest duration SQ treatment was supplemented for seven days via intraperitoneal (i.p.) injection at 200 mg/kg [47]. There were also studies involving SQ supplementation squalene for two to three weeks, which incorporated cardiomyopathy and cardiotoxicity tests [63,64].

In terms of source of squalene used, eight studies utilised pure SQ material obtained or purchased from a drug company [44,47,48,51,52,53,55,63], two studies used squalene in encapsulation form [45,49], nine studies used natural extracted squalene with the majority of them extracted from fresh shark liver (*n =* 6) [57,58,59,60,62], and the other remaining three studies used extracted SQ derived from amaranth grain [47,54,64]. However, there were three studies that did not mention the source of SQ used in their studies [46,50,56]. The majority of studies (*n =* 13) used lipid profile parameters to measure the effectiveness of SQ. Various parameters were evaluated and included in this review, comprising measurement of liver lipids (*n =* 6) [38,41,42,45,47,48], lipoprotein level(*n =* 5) [46,50,53,55,56], atheromatous/atherosclerotic area (*n =* 2) [55,56], lipid peroxidation status (*n =* 3) [58,59,61], and antioxidant properties (*n =* 5) [46,56,57,62,63]. Diagnostic marker enzymes such as ALT, AST, LDH, and CPK were measured in three studies [58,61,63]. There was only one study that measured HMG-CoA reductase activity [47].

## 4. Effects of Squalene on CVD for Animal Studies

A total of 18 animal studies were evaluated. Farvin et al. have five studies included in this paper with various parameters measured [57,58,59,60,62]. Supplementation of SQ extracted from shark liver oil to the isoprenaline-MI induced rats for 45 days demonstrated a significant decrease in total cholesterol and triglycerides in comparison to the control group. Meanwhile, the HDL-C in the group MI-supplemented with SQ showed significant increments when compared to that of the MI-control group [59]. Prior treatment of SQ in the isoprenaline-MI induced rats had significantly increased the antioxidant enzymes (GST and GPx) and antiperoxidative enzyme (CAT and SOD) levels [57]. During the follow-up study, it was reported that the pretreatment of 2% squalene in the diet to MI-induced rats was able to reduce the release of diagnostic marker enzymes such as ALT, AST, LDH, and CPK into the systemic circulation and to restore the membrane-bound ATPases (Na^+^K^+^ATPase and Ca^2+^ATPase) activity in the heart tissue at near-normal state [58]. The consecutive trial showed that the levels of protein and glycoprotein components (hexose and hexosamine) in plasma and heart tissue of MI-induced rats were significantly decreased compared to the control group. Additionally, in the presence of promoters such as ascorbic acid, ferrous sulphate (FeSO_4_), and tert-butyl hydroperoxide, SQ had successfully reduced lipid peroxidation level to near normalcy [60]. SQ supplementation has also been postulated to exert a protective effect on the endogenous antioxidants such as ascorbic acid and α-tocopherol as well as to restore the endogenous SQ content in the cardiac tissue [62].

In a much earlier study by Kritchevsky et al., orally fed SQ (regarded as a source of endogenous cholesterol) and cholesterol (regarded as exogenous cholesterol) were given to experimentally induce atherosclerosis rabbits. Supplementation with SQ showed an increase in liver weight and liver non-saponifiable materials but did not cause atherosclerosis [55]. Khor and Chieng (1997) fed a semisynthetic diet with 20% fat in palm oil triacylglycerol (POTG) to hamsters supplemented with 0.1% SQ for 45 days. There was a significant reduction of serum total cholesterol level and significant increase in liver total lipids and total cholesterol contents when compared to the POTG-control group [48]. In a study conducted by Shin et al. (2004), comparison between plant squalene (from amaranth grain) and animal squalene (from shark liver oil) revealed that amaranth squalene had caused a significant decrease in serum and liver lipids when compared to those of control and shark squalene-treated groups. Additionally, the amaranth squalene supplementation was able to significantly increase fecal excretion of cholesterol and bile acids as well as to inhibit the HMG-CoA reductase activity [47].

Dhandapani et al. in a 2007 study compared myocardial infarction (MI)-induced and non-MI induced (normal) rats supplemented with 2% SQ for two months. The pre-supplementation with SQ had significantly reduced the release of enzymes (ALT, AST, LDH, and CPK) from the myocardium into the systemic circulation when compared to MI-induced control rats. Moreover, the SQ supplementation had significantly reduced the lipid peroxides and significantly elevated GSH in comparison to the MI-induced control rats [61]. Guill´en et al. (2008) using Apo E knockout mice of both genders reported that 1% SQ supplemented in beverages had significantly decreased the plasma lipid profile in females whereas there were no changes in males. However, there was a significant reduction in liver weight and fat content in male mice supplemented with SQ when compared to the control group. Moreover, the male mice supplemented with SQ showed a significant decrease in size of atherosclerotic lesion [56]. Liu et al. (2009) reported a positive finding of SQ on blood pressure (BP) and obesity management in rats whereby supplementation of high SQ diet (1000 mg/kg) significantly lowered their BP, plasma cholesterol, triglycerides, glucose, and leptin compared to that of the control group [51]. Furthermore, Motawi et al. (2010) reported that SQ treatment to cardiotoxic-induced rats had caused normalization of nitric oxide (NO) and calcium ions (Ca^2+^) when compared to a cardiotoxic control group, showing a significant elevation in GPx activity and a reduction in cardiac GSH level [63]. A long-term SQ supplementation (11 weeks) at 1g/kg dose to male wildtype (WT), Apo E-deficient, and Apoa1-deficient C57BL/6J mice showed significant increments in HDL-C level independence of genetic backgrounds without affecting the total cholesterol concentration [46]. In fact, SQ supplementation for the same duration at much lower doses (0.25 g/kg) also demonstrated a significant increase in total cholesterol level when compared to the control group [46]. In a study by Preobrazhenskaya et al. (2015), SQ was seen able to significantly reduce the plasma lipid profiles (TC, LDL-C, TG, and free fatty acids) and to significantly increase HDL-C levels of doxorubicin-induced cardiomyopathy rats when compared to the control group [64].

In a recent study, rats supplemented with 2% SQ of total diet had decreased triglyceride levels in liver and epididymal white adipose tissue. They also reported that, when n-3 fatty acid precursors were available, the ratio of the long-chain n-3 fatty acid, DHA, was significantly increased in the SQ-fed group when compared to control [52]. Smith et al. (2000) supplemented 1% SQ to high-fat diet hamsters for four weeks duration, reporting a significant increase of cholesterol in VLDL + IDL when compared to the high-fat diet control group. Moreover, the SQ supplemented group had a significant increment in plasma triglyceride and LDL-C/HDL-C ratio [53]. Castro et al. (2013) reported similar negative findings in which lipid profile parameters were increased in the SQ-treated group. The fecal excretion of bile acids, liver weight, and degree of liver steatosis were significantly greater in the SQ-treated group as compared to the control group [54]. Zhang et al. (2002) also reported negative findings on SQ, where 0.05% and 0.5% of SQ supplementation were introduced to the high-fat diet in hamsters demonstrating an elevation in serum total cholesterol and triglyceride levels [44].

## 5. Effects of Squalene on CVD for Human Studies

There were three human studies included in this paper. Strandberg et al. measured metabolic variables of cholesterol during squalene feeding in patients with cerebrovascular or cardiovascular disorders with different degrees of hypercholesterolemia. SQ was supplemented in capsules (300 mg) taken three times daily for 30 days. The measurements including the level of squalene, cholesterol, and non-cholesterol sterols in serum were performed at baseline, 7 days, and 30 days. The results demonstrated that SQ administration had caused an unchanged level of serum triglyceride and cholesterol content but significantly increased serum levels of free and esterified sterol contents. In addition, the SQ feeding revealed an inconsistent effect on cholesterol absorption efficiency but significantly increased fecal excretions of cholesterol, its nonpolar derivatives, and bile acids [45].

Miettinen and Vanhanen conducted a human study to determine the role of SQ feeding in various amounts in rapeseed oil to hypercholesterolemic patients for a long-term period (nine to 15 weeks). Prior to the squalene treatment, the study had created six weeks of a pre-squalene period, where rapeseed oil (vehicle) was provided to the participants. Although involving a small study population (*n =* 18), the study revealed significant values in result. The reported result indicated that the total cholesterol level at week 15 (following nine weeks of SQ treatment) had significantly increased when 1 g SQ was added with rapeseed oil. Additionally, the addition of 1 g SQ to the diet had significantly increased the level of LDL-C, total triglycerides, and VLDL when compared to that of pre-squalene period. However, when a small dose of SQ (0.5g) was introduced to the diet starting from week 15 to week 21 (six weeks period), TC and LDL-C were reduced at rates of 83% and 19%, respectively. Moreover, the additional of 0.5 g SQ into the diet significantly decreased the levels of IDL-C, triglycerides, and phospholipids [50].

An intervention trial conducted by Chan et al. to determine the efficacy and safety of SQ formulations in 102 elderly patients with primary hypercholesterolemia demonstrated that the participants’ TC and LDL-C levels were significantly decreased when compared to baseline level and the placebo-treated group following 20 weeks of supplementation on 860 mg SQ/day [49]. Moreover, there was a minor reduction of triglycerides (5.3%) and a minor increase of HDL-C (1.8%). In terms of safety, the 860 mg dose for a 20-week period of SQ treatment was well tolerated with minor and infrequent side effects.

## 6. Discussion

### 6.1. Diseases/Conditions included in the Review

Main diseases/conditions included in this review consist of i) hyperlipidemia with eight animal studies and three human studies, ii) atherosclerosis with two animal studies, and iii) myocardial infarction with six animal studies. Other than that, studies related to cardiomyotoxicity and cardiomyopathy were also included with one animal study each.

Hyperlipidemia can be defined as an isolated elevation of cholesterol, isolated elevation of triglycerides, as well as elevation of both. However, since lipids are insoluble in plasma, they are transported in particles known as lipoproteins. Therefore, the classification of hyperlipidemia is done based on abnormalities of lipoproteins including LDL, HDL, VLDL, IDL, and chylomicrons [66]. High levels of lipoproteins, especially LDL, are a major contributor to the increased risk of atherosclerotic lesion formation, which initially begins with endothelial damage. The endothelial damage leads to the dysfunction of endothelial cells and therefore causes increased permeation of LDL particles through the vascular wall. LDL may accumulate within the vessel wall, trapped by a cellular matrix in the intima, and is then modified.

The modified LDL will be taken up via scavenger receptors on macrophages resulting in foam-cell formation. Accumulation of more lipid within the wall of the vessel causes smooth muscle cells to begin migrating into the lesion. The migration of these smooth muscle cells may ultimately encapsulate the newly formed plaque forming the fibrous plaque, which acts as the protector of the lesion, preventing the lipid core from being exposed to the vessel lumen. The atherosclerotic plaques can lead to the occlusion of vessels, causing decreased blood flow distally and leading to cardiac ischemia [67]. Eventually, an acute clinical event known as myocardial infarction (MI) characterized by a sudden ischemic death of myocardial tissue may occur due to thrombotic occlusion of a coronary vessel caused by a ruptured vulnerable plaque [68]. Therefore, it can be concluded that hyperlipidemia and atherosclerosis are the primary causes of myocardial infarction, which remain a leading cause of CVD death worldwide [69].

Cardiotoxicity is a condition in which there is damage to the heart muscle, which occurs due to chemotherapy drugs or other medications. Severe cardiotoxicity may lead to cardiomyopathy, and the injury to heart muscle may cause a disturbance in the heart’s pumping action as well as subsequent heart failure [70].

### 6.2. Animal Studies

Most animal studies showed positive effect of SQ on CVD. There was strong evidence for the benefits of squalene in various animal models using rats with 10 studies [47,51,57,58,59,60,61,62,63,64], mice with three studies [46,52,56], as well as hamsters and rabbits with one study each [48,55], indicating positive changes for parameters related to CVD. Based on the selected animal studies, the rat model has been the mostly utilized model due to the promising reproducibility and robust adaptation for pharmacological induction method of myocardial damage in rat hearts [71]. There were six studies that used isoprenaline injection to induce myocardial infarction in rats [57,58,59,60,61,62]. Isoprenaline induces cardiac myocyte necrosis, extensive left ventricular (LV) dilatation, and hypertrophy [71]. Other pharmacological inductions of heart damage used doxorubicin to induce cardiomyopathy [64] and cyclophosphamide to induce cardiotoxicity [63]. Both doxorubicin and cyclophosphamide are anticancer drugs with cardiotoxicity as their major adverse effect [72,73] and are commonly used to induce cardiotoxicity and heart failure (HF) in animal models [74,75,76,77]. Hypercholesterolemia is commonly induced by high fat or cholesterol diet. For instance, Shin et al. used as low as 1% cholesterol diet for four weeks to induce hypercholesterolemia in the rats [47]. In a study by Matos et al. (2005), 1% cholesterol diet has been observed to promote an increased LDL-C, to reduce HDL fractions, and to affect less hepatic function [78].

For animal study, there were three studies that reported negative findings of SQ in CVD using hamsters [44,53,54]. Generally, hamsters have an atherogenic lipoprotein profile with a great proportion of non-HDL forms of circulating lipoproteins. They possess receptor-mediated uptake of LDL lipoproteins, cholesteryl ester transport protein (CETP), exclusively hepatic production of apolipoprotein (Apo) B-100, and intestinal production of Apo B-48 [79,80,81]. Hence, they may quickly develop hypercholesterolemia when being fed with a cholesterol-rich diet, making hamsters an excellent species to study diet-induced atherosclerosis [82].

#### 6.2.1. Animal Studies Related to Hyperlipidemia 

Khor and Chieng (1997) have shown that low-level (0.1%) squalene had significantly lowered serum cholesterol levels in high-fat diet hamsters. Simultaneously, squalene supplementation has also significantly increased other lipids in the liver, showing the enhancement of cholesterol esterification in the liver [48]. This has postulated that the action of squalene to lower serum cholesterol may occur by enhancing cholesterol esterification in the liver.

Shin et al. (2004) revealed that SQ also caused significant decrease in serum TC and TG in hypercholesterolemic-diet induced rats. In the experiment, squalene extracted from amaranth plants showed better regulation of cholesterol-lowering effects compared to shark liver SQ. This might be due to the increased fecal elimination of cholesterol and bile acids as well as the inhibition of HMG-CoA reductase activity by amaranth SQ. A different source of squalene might act differently in the cholesterol biosynthesis pathway [47]. In an evidence-based review, plant-based diets have demonstrated greater cholesterol reductions compared to lean-meat containing diet [83]. This result might support the potential of plant SQ in cholesterol-lowering effect compared to animal SQ. Moreover, amaranth contains the highest amount of SQ (5942 mg/100 g) compared to other common plant oils such as olive oil (564  mg/100 g), soybean oil (9.9  mg/100  g), rice, wheat germ, grape seed oil (14.1  mg/100  g), and peanut (27.4  mg/100  g) [32].

Liu et al. (2009) demonstrated that high SQ diets (1000 mg/kg) had significantly lowered the cholesterol, triglycerides, and blood pressure in rats. This finding indicated the potential of SQ as an alternative treatment for hyperlipidemia and high blood pressure, thus preventing the occurrence of CVD [51]. Both high blood pressure and hyperlipidemia are among the most important risk factors for CVD [84].

Gaba´s-Rivera et al. [46] showed that SQ action in cholesterol-lowering effect was dose dependent, in which 11 weeks of 1 g/kg SQ treatment had significantly decreased TC and significantly increased the HDL-C. The increased HDL-C level was associated with augmented esterified cholesterol, indicated by the increased enzyme responsible for HDL-C known as lecithin: cholesterol acyltransferase (LCAT). LCAT plays a vital physiological role in the maturation of HDLs, modulating the conversion of HDL_3_ to HDL_2_ and thus collecting cholesterol from peripheral tissues and increasing HDL levels [85]. However, the supplementation of a lower squalene dose at 0.25 g/kg significantly increased TC without increasing HDL-C [44].

Kumar et al. (2016) reported that SQ supplementation in rats exhibited hypotriglyceridemic effect and significantly enhanced the ratio of EPA and/or DHA when their n-3 fatty acid precursors were available [52]. Both EPA and DHA are n-3 long-chain (LC) polyunsaturated fatty acids (PUFAs) and are associated with cardioprotective effects. Several mechanisms have been proposed to elucidate the reductions in CVD risk by n-3 LC PUFAs such as preventing cardiac arrhythmias, lowering plasma triglycerides, reducing blood pressure, decreasing platelet aggregation, and reducing inflammation [86]. Reduction in the risk of cardiovascular mortality after increasing the consumption of fatty fish or n-3 LC PUFA dietary supplements has been reported in several randomized controlled trials [87,88,89]. Thus, since SQ was able to elevate the n-3 LC PUFAs, particularly EPA and DHA that possess cardioprotective effects, this could possibly postulate the role of SQ to exert a hypotriglyceridemic effect.

However, the consequence of the high-fat diet provided in several experimental animal studies is not actually improved by administrating SQ, in which significant elevation in plasma cholesterol and triglyceride have been reported [44,53,54]. This might indicate that SQ may exert a hypercholesterolemic effect; thus, caution must be taken if squalene is routinely consumed as a health supplement.

#### 6.2.2. Animal Studies Related to Atherosclerosis

Kritchevsky et al. in their 1954 study has regarded dietary SQ as endogenous cholesterol since SQ has been presumed the most efficient precursor of cholesterol. In the study, SQ was not related to atherosclerosis but had resulted in increased liver weight and non-saponifiable material [55]. This condition was postulated to be associated with the SQ activity to enhance the cholesterol esterification activity in the liver and to consequently lower the serum cholesterol level [48], causing the atherosclerosis development not to be favored. According to Takeuchi and Yamamura, since the newly synthesized cholesterol is in the free form, esterification would increase before the release or degradation of cholesterol. Following esterification, the cholesteryl esters stored in the liver must be removed by biliary drainage and hepatic cholesteryl ester hydrolysis might increase [90]. Thus, enhanced fecal excretion of cholesterol and its nonpolar derivatives can be proposed as an alternative approach to regulate the lipid profile in SQ supplementation.

Guill´en et al. study confirmed that SQ modulates lesion development in a sex-specific manner, particularly better in male mice [56]. In a review by Insull (2009), it was stated that the process of lesion development is independent of sex but mainly dependent upon factors such as hypertension, tobacco smoking, diabetes mellitus, obesity, and genetic predisposition [91]. Although the lesion development in their study favored males, SQ supplementation had significantly decreased plasma cholesterol, triglycerides, and Apoa1 in females. However, this significant result was associated with low statistical power of prediction, which might be due to the low sample size or polymorphic genetic response with responder and nonresponder subjects [56]. Indeed, the number of female mice used in this study was much lower compared to male. In the male mice, SQ supplementation had significantly increased the hepatic Apoa5 mRNA expression with a high power of prediction. Theoretically, Apoa5 modulates triglyceride level by enhancing the catabolism of TG-rich lipoproteins and inhibits the rate of production of VLDL (the major carrier of TGs) [92]. However, the increased male Apoa5 mRNA expression might not be efficient to promote the actions on the plasma triglycerides. Based on all papers included in this review, this study was the only one introducing sex as a discriminating variable to uncover differential responses and even the potential of differential genetic responses in females [56].

#### 6.2.3. Animal Studies Related to Myocardial Infarction (MI)

Patients with acute MI are often associated with increased inflammation and oxidative stress. Free radicals play an important role in the pathogenesis of tissue damage in MI. Antioxidants may protect the body from damage caused by free radicals [93]. Under normal condition, there is a balance between tissue oxidant and antioxidant activity. The latter can be achieved by the antioxidant scavenger system, which includes enzymes like superoxide dismutase (SOD), catalase, glutathione peroxidase (GPx), and antioxidant vitamins (C, A, E, and other carotenoids) [94]. Farvin et al. (2004) revealed that SQ had significantly increased the activities of antioxidant enzymes (GPx and GST) and anti-peroxidative enzymes (CAT and SOD) in isoproterenol-induced MI rats antioxidant effect, probably due to the presence of isoprenoid unit in the SQ structure. In the study, the unpaired electrons in the hydroxyl radical (OH•) generated during isoproterenol-induced myocardial infarction may have been trapped for dismutation by free radical scavenging isoprenoid units [57]. In another study by Farvin et al. in 2009, it was found that SQ supplementation to MI-induced rats had significantly increased the endogenous antioxidants of vitamin C and vitamin E compared to MI-control rats [62]. These studies indicated that SQ supplementation exerts a deleterious effect of MI aberration in the antioxidant scavenger system and endogenous antioxidant vitamins in the MI-induced experimental rats.

In 1979, WHO recommended the panel of AST, LDH, and CPK for diagnosing acute MI [95]. However, the lack of specificity of AST by which it can be found in the liver, heart, skeletal muscle, brain, and kidneys has made it to be no longer used for the diagnosis of acute MI [96,97]. Currently, cardiac troponin (cTn) that is absent in non-myocardial tissues and is rapidly released into the blood following myocardial injury has been introduced as the standard [98]. Two animal studies that tested oral administration of SQ to MI-induced rats have shown improvement in cardiac markers (ALT, AST, LDH, and CPK) [58,61]. These animal studies did not just rely on the AST for diagnosing MI, but other parameters were also included. Both Farvin et al. (2005) [58] and Dhandapani et al. (2007) [61] revealed that SQ administration to MI-induced rats showed a reduction in lipid peroxidation and an elevation in GSH level. Decreasing the formation of lipid peroxidation products could be beneficial in limiting the deleterious effects of reactive oxygen species (ROS) in various pathological conditions including myocardial infarction [99]. Additionally, Farvin et al. (2005) [58] revealed that SQ supplementation exerted membrane-stabilizing action against isoproterenol MI-induced conditions by maintaining the activities of membrane-bound enzymes such as Na^+^/K^+^ ATPase and Ca^2+^ ATPase in heart tissues and mineral status (Na^+^, K^+^ and Ca^2+^) at near-normal levels. The activities of ATPases are closely associated with the plasma membrane and participate in the energy-requiring translocation of sodium, potassium, and calcium. The inhibition of Na^+^/K^+^-ATPase can activate the Na^+^–Ca^2+^ exchange mechanism in the myocardium for regulating the cellular calcium levels. The Ca^2+^ ATPase is the major active calcium transporter responsible for maintaining normal intracellular calcium level in various cells [100].

Farvin et al. in 2006 reported that SQ had significantly decreased the level of cholesterol, TG, free fatty acids, phospholipids, and LDL-C in MI-induced rats from the blood sampling performed at 24 h after the last MI induction [55]. Several previous studies have reported that, within 24 h of MI, most of the patients had high levels of TC, TG, and LDL-C and low levels of HDL-C [101,102,103]. This indicated that SQ possesses hypolipidemic property to improve the lipid profile in acute MI patients within 24 h onset of MI.

Farvin et al. (2007) [60] also revealed that SQ was able to maintain the antioxidant status of GSH levels and to reduce protein, hexose, and hexosamine levels in plasma and cardiac tissue, therefore resisting the isoproterenol-induced damage. Isoproterenol induces marked endocardial injury, which is associated with hypertrophy of surviving myocytes and an increase in myocardial fibrosis [104]. Glycoproteins such as hexose and hexosamine are important for stabilizing the tissues and for maintaining the structural stability of collagen fibrils [105]. In contrast, the aberrant glycoprotein components modulate ion channel activity and electrical signaling in the cardiac cell membrane [106]. Thus, SQ pretreatment exerts cardioprotective effect by preventing necrotic damage of the myocardial cell membrane through membrane-stabilizing action and antioxidant properties.

#### 6.2.4. Animal Studies Related to Cardiotoxicity and Cardiomyopathy

Motawi et al. revealed the improvement in cardiac markers (CPK, LDH, and AST) by the action of SQ in cardiotoxic induced rats [63]. Cyclophosphamide administration to the rats for cardiomyotoxicity induction decreased the level of antioxidant markers and elevated the level of cardiac biomarkers [107]. Other parameters measured in the trial, namely serum uric acid, creatinine, and urea levels, as well as cardiac antioxidant status and calcium levels could further support the positive findings of cardiotoxicity effect of SQ. Other parameters showed improvement levels when SQ was supplemented to the cyclophosphamide-cardiotoxic induced rats. The significant increase in their cardiac antioxidant status provided cellular defense against ROS to the cardiac cells. Apart from that, the antioxidant nature of SQ was observed later, where antioxidant enzymes (GPx and GR) showed marked increases in their activities [63]. Thus, this indicated the potential of SQ as a cardioprotective agent due to its ability to reverse the cyclophosphamide-induced cardiomyotoxicity effects.

Preobrazkaya et al. (2015) reported that 6% SQ supplementation in corn oil to doxorubicin-induced cardiomyopathy rats had significantly reduced the TC, LDL-C, TG, and free FA profiles in plasma. Meanwhile, the level of HDL-C was significantly higher following the SQ supplementation to the cardiomyopathy-induced rats [64]. The pathogenesis of cardiomyopathy of doxorubicin is complex and generally involves the accumulation of fat, hypercholesterolemia, and disturbance in lipoprotein metabolism [108]. Therefore, the supplementation of squalene can give a significant cardioprotection against doxorubicin-induced cardiomyopathy by the amelioration of lipid -lowering effect.

### 6.3. Human Studies

Generally, the data from animal experiments conducted prior to clinical work can inform the decisions about how treatments should be taken forward in clinical trials, for instance, in terms of animal extrapolation dose to human dose. However, any biased or imprecise results from animal experiments may result in the testing of biologically inert (not effective) or even harmful substances in clinical trials, thus exposing patients to unnecessary risks besides wasting the research fund [109].

Meanwhile, comparing the outcome of human and animal studies could be difficult since the translation of results from animals to humans might be hindered by various differences. Controlling environmental factors, food intake, and confounding factors might be possible in animal studies but impossible in human studies. The mechanisms of which SQ exerts its effects could be different in humans. For human studies, interaction between SQ and other nutrients, chemicals, and food sources must be clearly explored. Thus, differences between animal studies and human epidemiological studies in this review are therefore expected.

#### Human Studies Related to Hypercholesterolemia

All three human studies included in this review showed contradictory findings [45,50] and positive [49] effects of SQ in CVD. The human studies included in this review have tested SQ on hypercholesterolemia patients. Strandberg et al. (1990) [45] reported that SQ had no consistent effect on serum cholesterol level in a serial analysis of serum cholesterol with or without SQ. Additionally, the SQ feeding at 900 mg/day showed no changes in esterified and total cholesterol concentrations after seven to 30 days while significantly increased free cholesterol concentration in serum at day 7 of the measurement. Inconsistent result has been reported where long-term intake (nine weeks) of SQ at 1 g/day had mimicked a diet rich in SQ, resulted in elevated cholesterol precursor sterols, and thus raised the cholesterol level. However, a smaller dose of SQ (0.5 g/day) normalized the situation [50]. In the study, the large SQ intake (1 g/d) had stimulated increased kinetics of LDL apolipoprotein B, an atherogenic lipoprotein [110,111] that possibly contributes to the increased cholesterol synthesis. Relas et al. (2000) reported that dietary SQ had increased cholesterol synthesis [112], which might be supported by the fact that SQ (which is a precursor sterol) is partly converted to cholesterol in intestinal mucosa with a significant capacity to synthesize cholesterol [113]. In humans, intestinal absorption of SQ has been estimated to be around 60% [45]. It was also postulated that the newly absorbed SQ was converted to precursor sterols and further to cholesterol in the liver due to high postabsorptive SQ concentrations in VLDL [112].

A positive outcome was reported, indicating prolonged SQ consumption at 0.86 g/day for 20 weeks that has significantly reduced serum cholesterol (TC and LDL-C) and increased HDL-C levels [49]. The postulated mechanism of this anti-cholesterolemic property of SQ might be related to the downregulation of HMG CoA reductase activity through a feedback inhibition mechanism due to the enhanced SQ-derived synthesis of cholesterol. HMG CoA reductase is the rate-limiting enzyme of mevalonate pathway (cholesterol biosynthesis) [50]. There were many differences in the intervention period of SQ between the human studies, where Chan et al. [49] had the longest duration (20 weeks with 0.86 g/day) and followed by Miettinen and Vanhanen [50] (nine weeks with 1 g/day and six weeks with 0.5 g/day) and where the shortest duration was the study by Strandberg et al. [45], (7 to 30 days with 0.9 g/day). An appropriate intervention period should confirm the demonstration of efficacy and establishment of effect sustainability. This is because the human body might need a certain period of time to reach a stable effect on the endpoint or outcome measures for capturing significant biological effect [114,115]. Therefore, the prolonged intervention in the Chan et al. [49] study might be associated with better efficacy and sustainability of SQ treatments compared to the other two human studies.

## 7. Strengths and Limitations of this Review

The research on the effects of SQ in CVD is a promising area with important findings published in the last two decades, therefore making a critical review to be highly relevant. Our search identified 21 research articles in this paper, and we believe that this is the first critical review on this subject matter focusing on SQ and CVD. We have also included both animal studies and human studies in this review, which provided a better overview on the most recent and reliable evidence available. Many of the original articles included in this review measured various parameters, which could determine the mechanism of SQ actions. Nevertheless, this review has several limitations. Both animal and human studies included other agents (i.e., vitamin E and statins), and the effects may be compared to SQ. However, results were thoroughly screened to avoid misrepresentation of the results with other agents. The human studies included in this review used a limited number of participants (two studies with n < 20 and one study with *n =* 102). 

## 8. Recommendations

Based on the various study parameters, especially among the animal studies, it is thus important for any future trials to assess the effects of SQ with lipid profile parameter together with other related parameters. In the animal studies included, the source of SQ used was only limited to shark liver and amaranth oil. Despite the richest SQ content in shark liver oil, an alternative of other sources of SQ, especially plant-based, should be further explored in reducing the dependency to the animal-based SQ. To date, SQ can also be found in olive oil, palm oil, and wheat germ oil. In terms of human trials, an effort should be made in designing controlled human observational trials that will help in reducing the number of potential confounders in the analysis of the result. These measures will ensure that proper meta-analyses could be conducted in the future to give a clearer picture of the actual effect of SQ on CVD.

## 9. Conclusions

In this evidence-based review, one human study has reported a positive SQ outcome in CVD while the other two human studies revealed inconsistent results. Meanwhile, the majority of animal studies (15 out of 18 studies) have reported positive outcomes of SQ in CVD. Thus, the outcomes of the studies included in this review were heterogeneous.

For the potential of SQ in being used for cardiovascular-related diseases, it can be concluded that SQ, as the intermediate precursor of cholesterol, seemed to exert the hypocholesterolemic effect by inhibiting HMG-CoA reductase as a result of increased cholesterol synthesis by dietary squalene. Additionally, SQ employs cardioprotective effects from its antioxidant property due to its rich content of double bonds structure. The potentials possessed by SQ may benefit the healthcare providers and patients as well as researchers. The effects of SQ, especially plant-based, on lipid profile and other related parameters warrants further exploration. Additionally, controlled human observational trials should be performed to provide a stronger evidence. Due to the heterogeneous outcomes of the studies included in this paper and the potentials highlighted, we recommend further studies to be conducted to determine the net effect of SQ on CVD.

## Figures and Tables

**Table 1:** Characteristics of animal studies related to hyperlipidemia included in the review.

Study	Disease/ Condition	Study Type / SQ Source	Sample/ Population	Methodology	Results (SQ and CVD Only)	Comments/Outcomes
Study 1 Khor and Chieng 1997 [48]	Hyperlipidemia	Animal study Obtained from Sigma, St. Louis, MO, USA	Male Syrian hamsters (123 ± 2 g) The animals were randomly assigned into four groups.	The hamsters were fed with semi-synthetic diet containing 20% (w/w) of fat and a palm oil triacylglycerol (POTG) fraction isolated from commercial palm olein. They were divided into 4 groups: a) POTG b) POTG + SQ (POTG-SQ) c)POTG + SQ + pure tocotrienols (POTG-SQ-T3) d) POTG+ SQ and α-tocopherol (POTG-SQ- αT). The animals were fed accordingly for 45 days. SQ, T3, and α-T were supplemented at concentrations of 0.1%, 162 ppm, and 72 ppm, respectively. At the end of the treatment period, fasting blood samples were taken for the measurement of serum TC, HDL-C, TAG, and LDL-C. The livers were excised for measurement of liver lipids.	Serum: SQ-alone supplementation had significantly lowered (*p* < 0.05) serum TC levels as compared to the POTG group SQ-alone supplementation also lowers HDL-C, LDL-C, and TAG levels, but the reduction was not significant (*p* > 0.05) as compared to the POTG group. Liver: SQ-alone supplementation had significantly increased liver total lipids (*p* < 0.0l) and TC levels (*p* < 0.05). SQ-alone supplemented group had elevated the liver TAG level, while other liver lipids such as diacylglycerols (DAG), monoacylglycerols (MAG), free fatty acids (FFA), and phospholipids (PL) were not affected.	Squalene supplemented in short-term period (45 days) at a low level (0.1%) reduced serum TC. The ability of squalene to lower serum TC might be enhanced by cholesterol esterification activity in the liver.
Study 2 Shin et al. 2004 [47]	Hyperlipidemia	Animal study Amaranth SQ (in-house extraction) Shark liver SQ (obtained from Sigma, St. Louis, MO)	Male Sprague Dawley rats (110–130 g) Randomly divided into three groups	All rats were fed with 1% cholesterol diet for four weeks to induce hypercholesterolemia and were assigned into i) group 1: injected with saline (control) ii) group 2: injected with amaranth SQ (AS) iii) group 3: injected with shark liver SQ (SS) Both treatment groups, AS and SS, were injected at 200 mg/kg via intraperitoneal route for seven days prior to the sacrifice. Fecal samples were collected for the last 3 days for analysis of steroid excretion. Blood samples were analyzed for serum and liver lipids. The liver was excised to obtain liver microsomes for HMG-CoA reductase activity.	AS injection to the rats caused significant(*p* < 0.05) decrease in serum (TC and TG) and hepatic (cholesterol and TG) profiles compared to control and SS groups. Serum HDL-C levels in the AS group were significantly increased, resulting in a 45% reduction in atherogenic index. The AS group also significantly (*p<* 0.05) increased in fecal excretions of cholesterol and bile acid and slightly inhibited HMG-CoA reductase activity. However, these effects were not observed in SS-injected group.	Amaranth SQ (AS), which represents a plant source of SQ, exerts better hypolipidemic effects compared to the shark liver SQ (animal source of SQ). The authors concluded that the cholesterol-lowering effect of amaranth SQ might be mediated by the following: i) increased fecal elimination of steroids and ii) different sources of SQ (plant vs animal) that might affect cholesterol metabolism.
Study 3 Liu et al. 2009 [51]	Hyperlipidemia	Animal study Obtained from Healthy Nature Resource Inc	40 male Wistar rats (22 days old) Randomly divided into two groups (20 rats per group)	The two groups were fed with the following diet for four weeks: 1) control group received a diet without squalene and 2) SQ group was provided with high squalene diet (1000 mg/kg orally). When the rats were 51 days old (day 0 after the SQ withdrawal) and 75 days old (day 24 after the SQ withdrawal), ten rats from each SQ and control group were sacrificed. Blood samples were collected for measurements of leptin, glucose, cholesterol, and triglycerides. Tail arterial blood pressure (BP) and body weight were monitored weekly.	Following SQ feeding, BP and body weight gain were lower in the SQ group. BP was significantly lower from 47 days of age in the squalene-fed group compared to controls (*p* < 0.01). Plasma leptin, glucose, cholesterol, and triglycerides in SQ fed rats group were significantly lower compared to the control group at 51 and 75 days of age (*p* < 0.05).	Squalene may counteract the increase in body fat, BP, and levels of plasma leptin, glucose, cholesterol, and triglycerides. Squalene may act as an alternative treatment for clinical management of high blood pressure (BP) and obesity.
Study 4 Gabas-Rivera et al. 2014 [46]	Hyperlipidemia	Animal study The source was not disclosed.	Male wild-type (WT), Apo E-deficient and Apoa1-deficient mice on C57BL/6J genetic background Following baseline blood samples, groups with similar initial plasma cholesterol were established.	All mice were fed chow semi-purified diets and divided into 1) control groups: a) WT mice, *n =* 6; b) Apoa1-deficient mice, *n =* 7; and c) Apo E-deficient mice, *n =* 13 and 2) SQ-treated animals: a) WT mice received 1 g SQ/kg BW, *n =* 7; b) Apoa1-deficient mice received 1 g SQ/kg BW, *n =* 7; and c) Apo E-deficient animals were given two different doses of SQ: 0.25 g SQ/kg BW, *n =* 13 and 1 g SQ/kg BW, *n =* 14 The animals were fed the experimental diets for 11 weeks. Following the intervention, blood was sampled for measurement: a) plasma parameters TC, TG, HDL-C, nonesterified fatty acids, and aryl esterase; b) lipoprotein profiles VLDL, LDL, and HDL; and c) oxidative stress variable ROS levels in lipoprotein fractions.	Plasma parameters WT mice: The supplementation of SQ at 1 g/kg BW significantly increased HDL-C, nonesterified fatty acids, and aryl esterase activity when compared to the control group (*p* < 0.05). Apoa1-deficient mice: The supplementation of SQ at 1 g/kg BW significantly increased HDL-C and aryl esterase activity when compared to the control group (*p* < 0.05). Apo E-deficient mice: The supplementation of SQ at 1 g/kg BW significantly increased HDL-C when compared to the control and 0.25 g/kg BW SQ-treated groups (*p* < 0.05). The supplementation of SQ at 0.25 g/kg BW significantly increased total cholesterol when compared to the control and 1 g/kg SQ-treated groups (*p* < 0.01). Lipoprotein profiles WT mice: The administration of SQ at 1 g/kg BW induced an increase in HDL cholesterol that was accompanied by increased HDL phosphatidylcholine, whilst no changes in sphingomyelin and Apoa1 and redistribution of Apoa4 towards smaller HDL particles were observed. Apoa1-deficient mice: The administration of SQ at 1 g/kg BW did not have any effect on cholesterol distribution, decreased phosphatidylcholine, and increased Apoa4 in HDL particles and did not modify HDL sphingomyelin but decreased this phospholipid in LDL. Apo E-deficient mice: The SQ administration at either studied dose had little effect on cholesterol, esterified cholesterol, phosphatidylcholine, and sphingomyelin. However, it increased the presence of Apoa4 in HDL and decreased LDL Apoa4. Oxidative stress WT mice: The supplementation of SQ at 1 g/kg BW significantly reduced levels of ROS in isolated VLDL, LDL (*p* < 0.01), and HDL(*p <* 0.05) as well as plasma malondialdehyde (MDA) levels (*p* < 0.01) compared to the control group. Apoa1-deficient mice: The supplementation of SQ at 1 g/kg BW significantly reduced levels of ROS in isolated LDL (*p* < 0.01) and HDL (*p* < 0.05) compared to the control group. Apo E-deficient mice: The supplementation of SQ at 1 g/kg BW significantly reduced levels of ROS in isolated VLDL (*p* < 0.01) and HDL (*p* < 0.05) as well as plasma malondialdehyde (*p* < 0.01) compared to the control group. The supplementation of SQ at 0.25 g/kg BW significantly reduced levels of ROS in isolated VLDL (*p* < 0.01), HDL (*p* < 0.01), and LDL + HDL (*p* < 0.05) as well as plasma malondialdehyde (*p* < 0.01) compared to the control group.	SQ supplemented at a high dose (1 g/kg) for a long-term administration (11 weeks) significantly increased HDL-C level with independence of the genetic background without elevating TC level. SQ supplementation can modify HDL composition depending on the presence of Apo E and Apoa1. SQ supplementation can elicit an antioxidant action by decreasing oxidative stress level in lipoprotein fractions.
Study 5 Kumar et al. 2016 [52]	Hyperlipidemia	Animal study Obtained from Wako Pure Chemicals, Ltd., Osaka, JP	Obese/diabetic model KK-Ay mice (male, four weeks age) The mice were randomly divided into control and experimental groups (*n =* 7).	The groups were as follows: control fed with fat diet and test diet fed with fat diet contained 2% SQ. At the end of the experiment (4 weeks), all animals were sacrificed to collect blood and other organs. Measurement involved i) fatty acid profile and ii) TG, cholesterol levels in serum, liver, and epididymal white adipose tissue (WAT).	There was a significant decrease in the liver and epididymal WAT TG of SQ fed experimental groups as compared to control (*p* < 0.05). TG levels in serum and cholesterol levels in serum, liver, and epididymal WAT were not significantly different from control(*p* > 0.05). There was a significant increment in proportions of the long-chain n-3 fatty acid, DHA, in the squalene-fed group when compared to control (*p* < 0.05).	SQ influenced the lipid metabolism as seen by the TG levels and fatty acid profiles in the test diet fed to KK-Ay mice. SQ potentially exhibits a hypotriglyceridemic effect.
Study 6 Smith et al. (2000) [53]	Hyperlipidemia	Animal study Obtained from Sigma, St. Louis, MO	Thirty-six male adult F1B hamsters (170 to 250 g) During phase three, the animals were randomly assigned to three different diet groups (12 animals each).	This study was divided into three phases: phase 1 (acclimatization): the animals were fed with a normal chow diet; phase 2: the animals were fed with a high-fat diet for four weeks; and phase 3: the animals were randomly assigned to the following three groups for four weeks: i) group 1 continued with the same high-fat diet, ii) group 2 was fed a high-fat diet supplemented with 1% SQ, and iii) group 3 was fed a high-fat diet plus 0.5% β-sitosterol. At the end of each phase, major lipoproteins, namely chylomicrons, VLDL + IDL, and HDL were isolated and their concentrations were measured.	Cholesterol: During phase 3, both un-supplemented and SQ-supplemented groups (groups 1 and 2) showed significant increases in cholesterol content in VLDL + IDL (*p*< 0.01) compared with their phase 2 values. There were no significant changes observed in any of the groups on LDL-C and HDL-C levels. Triglycerides/ HDL and LDL-C ratios: During phase 3, group 1 and group 2 had comparable plasma triglyceride levels. The SQ-supplemented group (Group 2) significantly increased plasma triglyceride and LDL-C/HDL-C ratio in comparison to the level at phase 1.	Under this experimental condition, SQ supplementation at 1% of the total diet did not produce hypocholesterolemic and hypotriglyceridemic effects in the high-fat diet hamsters.
Study 7 Zhang (2002) [44]	Hyperlipidemia	Animal study Obtained from Sigma, St. Louis, MO	Thirty male Golden Syrian hamsters (95 ± 5 g) Divided into five groups (*n =* 6)	Each group was fed with one of the following diets for 4 weeks: 1. high-fat diet (HFD); 2. HFD + 0.05% pure SQ; 3. HFD + 0.1% pure SQ; 4. HFD + 0.5% pure SQ; and 5. HFD + 0.05% SQ-containing shark liver oil (SLO). At the end of the treatment, blood was sampled for serum lipid measurements. Livers, heart, and adipose tissue were collected for cholesterol measurement.	Elevation in serum TC in all groups supplemented with SQ (groups 2 to 5) In comparison to the control, serum TC was significantly (*p <* 0.05) increased in groups 2 and 4. A similar trend was observed for serum TG. Significant elevation in serum HDL-C in the 0.1% SQ and 0.5% SQ groups but not in the 0.05% SQ group as compared with the control hamsters No significant differences in serum non-HDL-C were observed among the five groups. SQ supplemented at 0.5% had significantly increased the cholesterol level in the liver and adipose tissue compared to the control. No differences in the cholesterol levels in the heart were observed among the five groups.	The investigators had concluded that SQ exerts a hypercholesterolemic effect at least in hamsters. Thus, caution must be exercised when SQ is routinely consumed as supplements.
Study 8 Castro et al. 2013 [54]	Hyperlipidemia	Animal study Amaranth oil (in-house extraction)	Forty-six male Golden Syrian strain hamsters, weanling (approximately 21 days), of sanitary standard type The animals were randomly divided into five groups.	Following seven days of adaptation, the rats were fed with commercial diet. Prior to further dietary regimen, six animals were randomly selected and fasted for 8 h before blood collection. The remaining 40 animals were divided into groups according to different diets and were fed for four weeks: i) control was fed a balanced diet containing 20% corn oil as the lipid source; ii) hypercholesterolemic was identical to the control group but contained 12% coconut oil, 8% corn oil, and 0.1% cholesterol as the lipid source; iii) Amaranth oil was identical to the hypercholesterolemic group but substitute corn oil with amaranth oil; and iv) squalene was identical to the hypercholesterolemic group but admixed with SQ in the ratio found in amaranth oil. At the end of the experiment, the animals were sacrificed for blood and liver collection. The following parameters were included: a) analyses in plasma (TC, TG, and HDL-C) and non-HDL-C (LDL-C + VLDL-C), b) analyses in the liver: liver weight and hepatic cholesterol concentration, c) histological analysis: haematoxylin and eosin, and d) analyses in the feces: cholesterol and bile acids.	There was a significant increase in all lipid profile parameters in the amaranth oil and SQ groups when compared to the control group (*p* < 0.05). Fecal excretion of bile acids was significantly greater in the amaranth oil and SQ groups as compared to the control group (*p* < 0.05). The liver weight was significantly increased in the SQ group compared to the control group (*p* < 0.05).	The consumption of amaranth oil and its SQ component did not exert a hypocholesterolemic effect in hamsters fed on a high-fat diet but promoted an increase in fecal excretion of bile acids.

VLDL: very-low-density lipoproteins; HDL: high-density lipoprotein; ROS: reactive oxygen species; IDL: intermediate-density lipoproteins; Apoa1: apolipoprotein a-1;CVD: cardiovascular disease; DHA: docosahexaenoic acid; KK-Ay: a cross between diabetic kk and lethal yellow; BW: body weight; LDL-C: low-density lipoprotein cholesterols; HDL-C: high-density lipoproteins cholesterols; TAG: triacylglycerol; TC: total cholesterol; TG: triglyceride; AS: amaranth SQ; SS: shark liver SQ; F1B: bio F1B; ApoE: apolipoprotein E; Apoa4: apolipoprotein A4.

**Table 2:** Characteristics of animal studies related to atherosclerosis included in the review.

Study	Disease/ Condition	Study Type/ SQ Source	Sample/ Population	Methodology	Results (SQ and CVD only)	Comments/Outcomes
Study 1 Kritchevsky et al. (1954) [55]	Atherosclerosis	Animal study Purchased from Distillation Products Inc, Rochester, NY, USA.	Male rabbits (1.5 to 2 kg) Divided into five groups	Rabbits were randomly divided into five groups as follows: group I: normal diet (N), group II: N + SQ, group III: N + SQ (14 weeks), group IV: N + cholesterol, and group V: N + SQ + cholesterol. The treatments were prepared at 3% of the total diet and were mixed with corn oil. All groups were maintained on the diets for seven weeks, except for group III (14 weeks duration). After the treatment, the livers were weighed and assayed for total non-saponifiable material, cholesterol, and SQ. The aortas were visually examined for atherosclerotic lesions and were graded on a 0 to 4 plus scale (in the order of increasing severity). Ultra-centrifugal examination of the serum for β-lipoproteins was also performed.	a) Atheromatous lesions: SQ-alone-supplemented groups (II and III) did not develop lesion and corresponded to those seen in group I. The cholesterol- alone (group IV) and SQ + cholesterol feeding (group V) showed the highest degrees of average atheroma. b) Liver weight and total non-saponifiable material: The liver weight of seven-week squalene supplementation (group II) increased markedly when compared to the normal group (group I). There was an increase in total non-saponifiable material in all groups (II–V) compared to the normal group, with a much greater increase in groups IV and V. c) Serum β-lipoproteins: The animals on the seven-week squalene diet showed no increase in their serum lipoproteins. As squalene feeding continued, there was an increase in the serum levels.	Supplementation of SQ may be regarded as a source of endogenous cholesterol and did not cause atherosclerosis. Exogenous cholesterol played a more important role in the development of atherosclerosis than endogenous cholesterol. SQ might increase liver weight and total non-saponifiable material; however, no development of atheroma was observed.
Study 2 Guill´en et al. 2008 [56]	Atherosclerosis	Animal study Source of SQ was not disclosed.	Homozygous ApoE knockout mice (aged 2 months) Divided according to sex (17 males, 15 females)	Both groups (male and female) were fed on a standard chow diet and were assigned into two groups with different beverages: i) SQ group, 1% (v/v) glycerol solution supplemented in squalene to provide a dose of 1 g/kg/day and ii) control group, glycerol solution (vehicle). Duration of treatment: Ten weeks At the end of the treatment, the mice were sacrificed for blood collection and heart excision. Measurement involved a) plasma lipid and lipoprotein, b) liver parameters, c) area of atherosclerotic lesion, d) antioxidant defenses (paraoxonase activity and isoprostane levels), and e) hepatic apolipoprotein.	One% SQ supplemented in beverage significantly decreased plasma cholesterol, triglycerides, and Apoa1 in females (*p* < 0.05), while there was no change in males. The liver weight was significantly decreased in males consuming SQ (*p* < 0.05), while this effect was not evident in females. Male mice receiving SQ showed a significant decrease in fat in the liver (*p <* 0.05). Males receiving SQ showed a significant decrease in the lesion (*p <* 0.01), while female mice showed no change in their lesion area. In males, there was a high statistically significant direct correlation (*r*s = 0.81; *p* < 0.000) between hepatic weight and plasma triglycerides. A statistically significant direct association (*r*s = 0.64; *p* < 0.02) was also observed between the lesion area and hepatic fat accumulation. SQ intake significantly decreased plasma levels of 8-isoprostaglandin F2 in both sexes (*p <* 0.05). No significant change in hepatic *Apoa1* mRNA levels in both sexes Male mice receiving SQ showed significantly increased *Apoa5* expression (*p <* 0.01).	The administration of SQ modulates lesion development in a sex-specific manner. The accumulation of hepatic fat by the liver is highly correlated with lesion progression in males. SQ administration could be used as a safe alternative to alleviate atherosclerosis and hepatic steatosis, particularly in males.

**Table 3:** Characteristics of animal studies related to myocardial infarction included in the review.

Study	Disease/ Condition	Study Type/ SQ Source	Sample/ Population	Methodology	Results (SQ and CVD only)	Comments/Outcomes
Study 1 Farvin et al. 2004 [57]	Myocardial infarction	Animal study Isolated from fresh shark liver	Wistar strain male albino rats (100–120 g) Divided into four groups (six rats per group)	Following 7-day acclimatization, the rats were divided according to groups, were fed on a standard diet with added oils for 45 days, and were injected with saline or isoproterenol for 2 days. Isoproterenol was used to induce myocardial infarction in rats. Group I (control): 2% coconut oil and injected with saline Group II (control): 2% SQ and injected with saline Group III: 2% Coconut oil and injected with isoproterenol Group IV: 2% SQ and injected with isoproterenol At the end of the experiment (24 h after last isoproterenol injection), the rats were sacrificed for blood collection and heart excision. Measurement involved a) antioxidant enzymes GPx and GST, and b) anti-peroxidative enzymes CAT and SOD.	The prior treatment with SQ had significantly increased the activities of antioxidant enzymes (GPx and GST) and anti-peroxidative enzymes (CAT and SOD) in the heart tissue of group IV as compared to group III isoproterenol-induced myocardial infarcted rats (*p <* 0.001). The normal rats receiving SQ alone (group II) did not show any significant change in comparison to the control (group I).	SQ supplementation possesses a cardioprotective effect due to their antioxidant property. No adverse effect following a low dose of SQ supplementation (at 2%)
Study 2 Farvin et al. 2005 [58]	Myocardial infarction	Animal study Shark liver oil of *Centrophorus sp.* caught in Andaman waters	Wistar male albino rats (100–120 g) Divided into four groups (six rats per group)	All rats were fed on a standard diet with added oils for 45 days and were injected with saline or isoproterenol for 2 days according to the groups. Isoproterenol was used to induce myocardial infarction in rats. Group I (control): 2% coconut oil and injected with saline Group II (control): 2% SQ and injected with saline Group III: 2% coconut oil and injected with isoproterenol Group IV: 2% SQ and injected with isoproterenol At the end of the experiment, (24 h after last isoproterenol injection), the rats were sacrificed for blood collection and heart excision. Measurement involved a) diagnostic marker enzymes, b) membrane-bound ATPase (Na^+^, K^+^ ATPase, and Ca^2+^ ATPase) activities and mineral status (sodium, potassium, and calcium), and c) lipid peroxidation and GSH	The pretreatment of 2% SQ in the diet had significantly reduced the release of diagnostic marker enzymes (ALT, AST, LDH, and CPK) into the systemic circulation as compared to group III rats (*p <* 0.001). The pretreatment of 2% SQ in the diet also had significantly counteracted the isoproterenol-induced lipid peroxidation and maintained the level of GSH at near normalcy in group IV rats as compared to group III animals (*p <* 0.001). SQ supplementation exerted membrane-stabilizing action against isoproterenol-induced myocardial infarction by maintaining the activities of membrane-bound ATPase (Na^+^, K^+^ ATPase, and Ca^2+^ ATPase) activity in the heart tissue and the mineral status (sodium, potassium, and calcium) in plasma and heart tissue at near-normal levels.	The cardioprotective effect of SQ might be contributed by the antioxidant property and membrane-stabilizing action.
Study 3 Farvin et al. 2006 [59]	Myocardial infarction	Animal study Isolated from fresh shark liver	Male Wistar strain albino rats (120–150 g) Divided into four groups (six rats per group)	All animals were fed on a standard diet with added oils for 45 days and injected with saline/isoproterenol for 2 days according to the groups. Isoproterenol was used to induce myocardial infarction (MI) in rats. Group I (control): 2% coconut oil and injected with saline Group II (control): 2% SQ and injected with saline Group III: 2% coconut oil and injected with isoproterenol Group IV: 2% SQ and injected with isoproterenol At the end of the experiment, (24 h after last isoproterenol injection), the rats were sacrificed for blood collection and heart tissue excision Measurement involved a) lipid parameters in plasma cholesterol, triglycerides, free fatty acids, phospholipids, LDL-C, and HDL-C; b) lipid parameters in heart tissue cholesterol, triglycerides, free fatty acids, and phospholipids; and c) lipid peroxides in plasma and heart tissue.	For normal rats (group I vs group II), SQ supplementation to the normal rats (group II) had significantly increased plasma HDL-C levels compared to the group I normal control rats (*p <* 0.01). Plasma lipid peroxidation in group II (normal squalene-fed rats) showed a slight decline as compared with group I normal control animals (*p <* 0.05). For MI-induced rats (group III vs group IV), both plasma and heart tissue levels of cholesterol, TG, free fatty acids, phospholipids, and LDL-C in MI-induced rats in group IV were significantly decreased in comparison to that of group III (*p <* 0.001). In contrast, the HDL-C level in group IV had also significantly increased compared to Group III. Both plasma and heart tissue lipid peroxidation in group IV MI-squalene fed were significantly decreased when compared with group III MI-control rats (*p <* 0.001).	The pre-administration of 2% SQ for 45 days prevents the symptoms of isoprenaline-induced myocardial infarction in rats. The cardioprotective effect of SQ might be related to its ability to inhibit lipid accumulation by i) hypolipidemic property and ii) free-radical scavenging ability against isoprenaline-induced lipid peroxidation.
Study 4 Farvin et al. 2007 [60]	Myocardial infarction	Animal study Isolated from fresh shark liver	Twenty-four Wistar strain male albino rats (120–150 g) Divided into four groups of six rats per group	All rats were fed on a standard diet with added oils for 45 days and were injected with saline or isoproterenol for 2 days according to the groups. Isoproterenol was used to induce myocardial infarction in rats. Group I (control): 2% coconut oil and injected with saline Group II (control): 2% SQ and injected with saline Group III: 2% coconut oil and injected with isoproterenol Group IV: 2% SQ and injected with isoproterenol At the end of the experiment, (24 h after last isoproterenol injection), the rats were sacrificed for blood collection and heart tissue excision. Measurement involved a) protein content, b) hexose and hexosamine content, c) lipid peroxidation in the presence of promoters (ascorbic acid, ferrous sulphate, and tert-butyl hydroperoxide), and d) GSH	The pretreatment of 2% SQ (group IV) had significantly decreased the level of protein, hexose, and hexosamine in both plasma and heart tissue (*p <* 0.05). The group had also significantly increased in the level of GSH and maintained it to near normalcy (*p <* 0.05). In the presence of promoters (ascorbic acid, ferrous sulphate, and tert-butyl hydroperoxide) in the heart tissue, group IV (MI-SQ) had significantly decreased (*p <* 0.05) lipid peroxidation levels when compared to the control group (group III MI-control rats). The normal rats receiving squalene (group II) showed a significant change (*p <* 0.05) for the level of protein and hexose in the heart tissue when compared with normal control rats (group I).	The pretreatment of SQ might exert cardioprotective effects by preventing isoprenaline-induced necrotic damage to the myocardial cell membrane by its membrane-stabilizing and antioxidant properties.
Study 5 Dhandapani et al. 2007 [61]	Myocardial infarction	Animal study Shark liver oil *Centrophorus* sp. caught in the Andaman waters	Forty-eight Wistar strain male albino rats (120–150 g) Divided into four groups of 12 rats per group	The rats were fed on commercial feed added with the following oils at a 2% level for 60 days: group 1: coconut oil, group 2: SQ, group 3: PUFA concentrate, and group 4: SQ + PUFA concentrate. After 60 days, each group was further subdivided into eight groups of six rats per group: 1) groups 1a and 1b; 2a and 2b; 3a and 3b; and 4a and 4b, were i.p. injected with only saline for two days (control animals) and 2) groups 1b, 2b, 3b, and 4b rats were i.p. injected with isoprenaline for two days to induce myocardial infarction. At the end of the experimental period, the rats were sacrificed for blood collection and heart excision. Measurement involved 1) diagnostic marker enzymes (ALT, AST, LDH, and CPK) and 2) lipid peroxides (LPO), reduced glutathione (GSH), and antioxidant enzymes.	Pre-supplementation with SQ alone (Group 2b) had significantly reduced the release of these enzymes (ALT, AST, LDH, and CPK) from the myocardium into the systemic circulation as compared to group 1b isoprenaline-administered rats (*p <* 0.001). Pre-supplementation with SQ alone (group 2b) had significantly reduced the LPO (*p <* 0.001) and significantly elevated GSH and antioxidant enzymes when compared to group 1b isoprenaline-administered rats (*p <* 0.001). Th normal SQ-fed rats (group 2a) showed no significant changes when compared to normal control rats (group 1a) rats.	Supplementation of SQ significantly counteracts the isoprenaline-induced elevations in the levels of diagnostic marker enzymes and LPO and is able to maintain the level of antioxidant enzymes at near normalcy. The administration of SQ may exert significant cardioprotection against isoprenaline intoxication.
Study 6 Farvin et al. 2009 [62]	Myocardial infarction	Animal study Shark liver oil *Centrophorus* sp. caught in the Andaman waters	Male Wistar strain albino rats (120–150 g) Divided into four groups of six rats per group.	All animals were fed on a standard diet with added oils for 45 days and injected with saline/isoproterenol for 2 days according to the groups. Isoproterenol was used to induce myocardial infarction in rats. Group I (control): 2% coconut oil and injected with saline Group II (control): 2% SQ and injected with saline Group III: 2% coconut oil and injected with isoproterenol Group IV: 2% SQ and injected with isoproterenol At the end of the experimental period (24 h after the last isoproterenol injection), the animals were sacrificed for blood collection and heart tissue excision. Measurement involved 1) ascorbic acid, 2) alpha tocopherol, and 3) endogenous squalene content.	Prior administration of 2% SQ in the diet (group IV) significantly increased the endogenous antioxidants (vitamin C and vitamin E) compared to MI-control rats (group III) (*p <* 0.001). The administration of 2% SQ in the diet also significantly restored the membrane-bound SQ content in the heart tissue compared to MI-control rats (group III) (*p <* 0.001).	SQ supplementation potentially exerts a deleterious effect of isoprenaline-induced aberration in endogenous antioxidant vitamins in experimental rats. SQ may exert cardioprotective effect via the ability to counteract free radicals by its antioxidant nature or membrane-stabilizing action.

**Table 4:** Characteristics of animal studies related to cardiotoxicity and cardiomyopathy included in the review.

Study	Disease/ Condition	Study Type/ SQ Source	Sample/ Population	Methodology	Results (SQ and CVD only)	Comments/Outcomes
Study 1 Motawi et al. 2010 [63]	Cardiotoxicity	Animal study Purchased from Sigma Chemicals Company, St. Louis, MO, USA	Male Wistar albino rats (170–200 g) Randomly divided into four groups: group I (*n =* 18), group II (*n =* 20), group III (*n =* 20), and group IV (*n =* 17).	Group I acted as the vehicle-treated control. The remaining three groups received a single intraperitoneal injection of 200 mg/kg BW cyclophosphamide (CP) to induce toxicity. Two of these groups received either DL-alpha-lipoic acid (LA) (35 mg/kg BW) or SQ (0.4 mL/rat) orally seven days before and seven days after CP injection. At the end of the experiment, the rats were sacrificed for blood collection and heart excision. Measurement involved 1) cardiac markers CPK, LDH, and AST; 2) serum TAC level; and 3) GPx and GR activities, levels of GSH, MDA, NO, and Ca^2+^ in the heart of CP-administered rats.	SQ treatment caused normalization of the levels of NO and Ca+2, a significant increase in the activity of GPx (*p* < 0.01), and decreases in the levels of cardiac GSH (*p* < 0.01) and serum uric acid (*p* < 0.001) when compared with the CP group. Hearts from SQ-treated rats showed nearly normal architecture of the heart with mild focal hemorrhage between myocardial bundles. The SQ-treated group had a significant decrease in all cardiac markers compared to the control group	SQ was able to attenuate the pathological alterations in the heart of CP-induced cardiotoxicity. This may highlight the efficacy of SQ as cytoprotectants in CP-induced cardiotoxicity.
Study 2 Preobrazhenskaya et al. 2015 [64]	Cardiomyopathy	Animal study Amaranth oil	Male albino rats (180–200 g) Randomly divided into six groups (seven rats per group)	All rats received daily gavage according to the groups for three weeks. Group 1: amaranth oil at 0.25 mL/kg Group 2: SQ 6% in corn oil Group 3: vehicle only (refined corn oil) Group 4: amaranth oil at 0.25 mL/kg Group 5: SQ 6% in corn oil Group 6: vehicle only (refined corn oil) Groups 4–6 were intraperitoneally (i.p) injected with 15 mg/kg doxorubicin in six injections over two weeks for cardiomyopathy induction, while control animals in groups 1–3 were injected with physiological saline solely. At the end of the experiment, animals were sacrificed and blood was sampled for the determination of plasma lipid profile: LDL-C, HDL-C, TC, TG, free FA, and phospholipids levels.	SQ supplementation to the cardiomyopathy-induced group (group 5) significantly reduced the TC, LDL-C, TG, and free FA profiles in plasma (*p <* 0.05) compared to the control group induced with the cardiomyopathy group (group 6). The level of HDL-C was significantly higher in group 5 compared to group 6(*p <* 0.05). All changes of plasma lipid profiles had a tendency for normalization but did not revert them to the normal level.	SQ supplementation at a low dose exerts a protective role against doxorubicin-induced cardiomyopathy in rats but did not revert them to the normal level.

**Table 5:** Characteristics of human studies included in the review.

Study	Disease/Condition	Study Type/ SQ Source	Sample/ Population	Methodology	Results (SQ and CVD Only)	Comments/Outcomes
Study 1 Strandberg et al. 1990 [45]	Cerebrovascular and cardiovascular disorders with different degrees of hypercholesterolemia	Human study SQ capsule The source was not disclosed	Fifteen patients (8 males and 7 females) of cerebrovascular and cardiovascular disorders Prior to the study, the patients were on a standard hospital diet for several weeks with the mean cholesterol and SQ intakes as 300 and 7 mg/day, respectively.	The patients were divided into two groups: i) SQ (*n =* 9), administered in capsules (300 mg) three times daily with meals, in which three subjects were fed for a minimum period (one-week) and six subjects were fed for an extended period (30 days), and ii) cholestyramine group (*n =* 6), administered 8 g of cholestyramine resin four times daily with meals. Blood was measured in intervals: basal, 7 days, and 30 days: i) cholesterol, SQ, and non-cholesterol sterol in serum and ii) cholesterol, SQ, and precursor sterol concentrations in different lipoproteins.	Fecal analysis showed that approximately 60% of dietary squalene was absorbed. Serum triglyceride and cholesterol contents were unchanged. Serum SQ levels were increased 17 times. The SQ feeding significantly increased serum levels of free and esterified methyl sterol contents (*p <* 0.05), while elevations of free and esterified cholesterol and lathosterol levels were inconsistent. The SQ feeding had no consistent effect on absorption efficiency of cholesterol yet significantly increased the fecal excretions of cholesterol, its nonpolar derivatives, and bile acids (*p <* 0.05).	SQ supplementation can increase cholesterol synthesis; however, no association was found with the consistency increment of serum cholesterol level.
Study 2 Miettinen and Vanhanen 1994 [50]	Hypercholesterolemia	Human study Source of SQ was not disclosed	Eighteen male subjects mean (± SE) age: 50 *±* 3 y, with basal serum concentrations of cholesterol > 6 mmol/L and triglycerides < 2.5 mmol/L.	This study had four periods as follows: 1) baseline: home diet (*n =* 18); 2) rapeseed oil (*n =* 18), 6 weeks: 50 g of daily dietary fat intake was replaced with 50 g of a rapeseed oil mayonnaise per day for six weeks; 3) rapeseed oil (*n =* 8) or rapeseed oil + 1 g SQ/d (*n =* 10), 9 weeks (at week 15): ten subjects consumed rapeseed oil mayonnaise with 1 g SQ while the control group consumed rapeseed oil without SQ for nine weeks; and 4) rapeseed oil (*n =* 8) or rapeseed oil plus 0.5 g SQ/d (*n =* 10), 6 weeks (at week 21): ten subjects consumed rapeseed oil mayonnaise with 0.5 g SQ while the control group consumed rapeseed oil without SQ for six weeks. Fasting blood samples were collected for serum lipid (cholesterol, TG, phospholipids, apolipoprotein B, and HDL lipoprotein). Serum precursor sterols were also measured.	The addition of 1 g SQ to the diet at week 15 showed a significant increase in total cholesterol when compared to rapeseed oil-consuming only at week 6 and control rapeseed oil at week 15 and week 21 (*p* ≤ 0.05). The addition of 1 g SQ to the diet at week 15 showed significant increments (*p* ≤ 0.05) in LDL-C, total TG, and VLDL when compared at week 6 (rapeseed-given only). The addition of 0.5 g SQ to the diet at the 4th period showed a significant reduction in IDL cholesterol, triglycerides, and phospholipids (*p* ≤ 0.05). The reduction of SQ intake to only 0.5 g/d decreased the serum lipid concentrations to the rapeseed oil control and pre-squalene concentrations. Serum precursor sterols were significantly increased (*p <* 0.05) when compared at week 6 (rapeseed-given only).	A long-term (9 weeks) and large-amount SQ intake (1 g/d) might cause increased total cholesterol in serum due to augmented number of LDL particles with low surface lipids (free cholesterol and phospholipids) Small SQ doses (0.5 g/d) did not increase total cholesterol, which might be related to inhibition of endogenous pre-SQ cholesterol synthesis, which does not affect the LDL receptors or serum cholesterol. Long-term use of SQ resulted in the adaptation of cholesterol metabolism.
Study 3 Chan et al. 1996 [49]	Hypercholesterolemia	Human study, double-blind, randomized, placebo- controlled trial SQ capsule (Goldian Co., Singapore)	One hundred and two elderly (age > 65) patients with primary hypercholesterole-mia (TC > 250 mg/dL, TG < 300 mg/dL) were randomly assigned. The patients were enrolled after a three-month dietary intervention with the American Heart Association (AHA) Step I Diet. Patients with homozygous familial hypercholesterolemia, types I or III–V; hyperlipoproteinemia; cardiovascular; renal; hepatic; gastrointestinal; or metabolic diseases and malignancies were excluded.	Prior to randomization, any previous treatment with lipid-lowering drugs was discontinued for two months and a single-blind placebo lead-in period of four weeks was administered along with the dietary treatment. All patients then start consuming one of the following treatments with evening meal for a 20-week period: 1) 10 mg pravastatin; 2) two capsules (430 mg each) of SQ; 3) a combination of pravastatin and SQ; or 4) matching placebo. Levels of TC, HDL-C, and TG were determined at baseline after the fourth week lead-in placebo treatment and again after 4, 8, 12, and 20 weeks of the active treatment. LDL-C levels were determined by calculation. During each visit, blood was measured for concentrations of lipids and lipoproteins and for liver function and creatine kinase.	Supplementation of SQ 860 mg/day for 20 weeks had significantly decreased TC and LDL-C levels when compared to the baseline level and placebo group (*p <* 0.05). Supplementation of squalene 860 mg/day for 20 weeks had decreased TG levels by 5.3% while increased the HDL-C level by 1.8%.	Dietary squalene appeared to exert hypocholesterolemic activity. The authors proposed that this effect was due to the downregulation of HMG-CoA reductase activity by the enhanced squalene-derived synthesis of cholesterol.

## Abbreviations

Apo A	apolipoprotein A
Apo E	apolipoprotein E
Apoa1	apolipoprotein a-1
Apoa5	apolipoprotein a-5
ALT	alanine aminotransferase
AST	aspartate transaminase
BW	body weight
Ca+2	calcium ions
Ca2+ ATPase	calcium-dependent adenosine triphosphatase
CAT	catalase
CP	cyclophosphamide
CPK	creatine phosphokinase
CVD	cardiovascular disease
DHA	docosahexaenoic acid
EPA	eicosapentaenoic acid
FA	fatty acids
GPx	glutathione peroxidase
GR	glutathione reductase
GSH	reduced glutathione
GST	glutathione s-transferases
HDL-C	high-density lipoproteins cholesterols
HFD	high-fat diet
HMG-CoA	3-hydroxy-3-methylglutarylcoenzyme A
i.p.	intraperitoneal
IDL	intermediate-density lipoproteins
KK-Ay	a cross between diabetic kk and lethal yellow
LDH	lactate dehydrogenase
LDL	low-density lipoproteins
LDL-C	low-density lipoprotein cholesterols
LPO	lipid peroxides
MDA	malondialdehyde
MI	myocardial infarction
mRNA	messenger RNA
N	normal diet
Na^+^ /K^+^ ATPase	sodium potassium-dependent adenosine triphosphatase
NO	nitric oxide
POTG	palm oil triacylglycerol
PUFA	polyunsaturated fatty acids
SOD	superoxide dismutase
SQ	squalene
TAC	total antioxidant capacity
TAG	triacylglycerol
TC	total cholesterol
TG	triglyceride
TL	total lipids
VLDL	very-low-density lipoproteins
WAT	white adipose tissue
WHO	World Health Organization
